# A Qualitative, Small-Sample Study of Employment Challenges for People with Disabilities in Saudi Arabia

**DOI:** 10.3390/healthcare12030346

**Published:** 2024-01-30

**Authors:** Mohaned G. Abed, Lowai G. Abed, Todd K. Shackelford

**Affiliations:** 1 Department of Special Education, Faculty of Educational Graduate Studies, King Abdulaziz University, Jeddah 21589, Saudi Arabia; mabed@kau.edu.sa; 2 Department of Communication and Media Technology, College of Social Sciences and Media, University of Jeddah, Jeddah 21493, Saudi Arabia; lgabed@uj.edu.sa; 3 Department of Psychology, Oakland University, Rochester, MI 48306, USA

**Keywords:** employment, people with disabilities, employers, challenges, discrimination, workplace, Saudi Arabia

## Abstract

Many people live with disabilities and many countries worldwide are acting to provide people with disabilities opportunities to find and sustain gainful employment. Notwithstanding, people with disabilities still do not have the same access to employment as their counterparts without disabilities. Although some research has investigated these issues in Western countries, very little research has investigated these issues in the Middle East, in general, and in Saudi Arabia, in particular. The aim of the present study is to gain an initial understanding of the employment experiences of people with disabilities and prospective employers of people with disabilities in Saudi Arabia. We identify several potential obstacles preventing people with disabilities from securing and maintaining gainful employment in Saudi Arabia. We secured qualitative data from semi-structured interviews with five prospective employers and five individuals with disabilities in Saudi Arabia. We focused on identifying barriers to employment. The impact of demographic factors such as age, gender, and education on employment opportunities was also examined. The results suggest several practical conclusions and recommendations relating to improving and enhancing opportunities for employment for people with disabilities in Saudi Arabia.

## 1. Introduction

For individuals to meet their physiological, social, and economic needs, they need to have access to employment opportunities [1]. The ability to work is a crucial factor in relation to wealth and well-being, and for people with disabilities, work plays an important role regarding their capacity to participate in and contribute to society [1]. Around one billion people, or 15% of the world population, live with moderate to severe disabilities [2]. However, there is no consensus with regard to what the term “disability” means [3]. Much of the research around the world uses the definition provided by the World Health Organization [2], which defines disability as a physical or psychological impairment impacting the ability to function and accomplish tasks in the same manner as the rest of the population within the same age group.

Securing gainful employment is a leading challenge faced by many people across the world. For those with disabilities, this challenge is greater still, leading to them remaining unemployed for longer than people without disabilities. In pursuit of independence, people with disabilities typically work hard to secure gainful employment and thereby contribute to the development of their communities [4]. For many people with disabilities, however, achieving independence is a struggle. This is because even though they may want to develop their knowledge and master skills, their efforts are sometimes hampered by discrimination associated with their disability [5]. In consequence, advocates have contended that countries should enact laws to eliminate employment discrimination against people with disabilities. Relying on legislation alone is not enough to address the challenges of securing employment for people with disabilities, suggesting that countries should enact training and education programs to improve employment opportunities for people with disabilities [6]. Consistent with these policy recommendations, Saudi Arabia is planning for and applying practices and initiatives linked to training and education so that, by 2030, the country will have decent work and pay for all, including people with disabilities (see below for additional details and references). It is against this background that the present study seeks to identify challenges related to discrimination against people with disabilities in the Saudi Arabian employment context. We also explore strategies that might be implemented to promote equal opportunities and create inclusive work settings in Saudi Arabia.

### 1.1. Disability Status in Saudi Arabia and the Arab Region (Data and Percentages)

**Figure 1:**
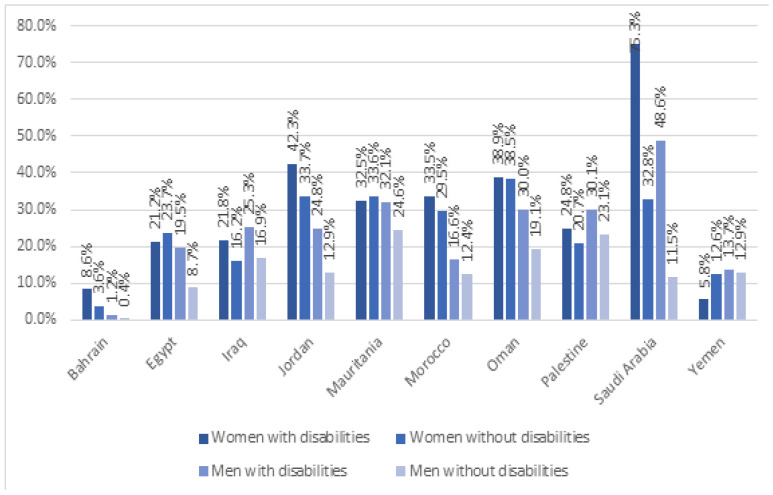
Shows the rate of unemployment among different genders with disabilities and those without disabilities in the Arab region aged 15 and above [8].

Since this study focuses on Saudi Arabia, highlighting statistical data and percentages relating to disability provides context to the study. Moreover, the data will provide insights into the qualitative analysis found in this study and provide quantitative context to the issue. While few studies and little research have been done to this end, we researched the entire Arab region, which we found useful for comparative reasons. More sourcing led us to Saudi Arabian government websites that could provide current and more accurate information concerning employment rates, percentages, and the level of discrimination faced. Foremost, we begin by defining disability based on the Saudi Arabia Government website, which refers to disability as “Any person who has a complete or partial deficiency stably. The deficiency can be physical, sensory, mental, communicative, educational, or psychological capabilities” [7]. Therefore, a person with a disability can have visual and hearing impairments, a mental disability, physical and motor impairment, learning challenges, speech and speech disorders, and behavioral and emotional disorders, as well as autism [8]. In Saudi Arabia, the percentage of people with disabilities constituted about 7.2% of the entire population of the Kingdom [7]. Based on the Statistics Authority, the number of people with disabilities in the Kingdom is shown in Table 1:

The rate of unemployment among individuals with disabilities in the Kingdom of Saudi Arabia, just like elsewhere in the world, is high, and the same is true for cases of discrimination. However, this varies depending on where one is located (rural or urban region) and their level of education. Moreover, the unemployment rate for people with disabilities is high for both genders compared to their non-disabled counterparts, but is even more so for females [7]. Figure 1, below, highlights the rate of unemployment in the Arab region, and the most extreme differences are seen in Saudi Arabia, where the unemployment rate for women with disabilities (75.3%) is two times higher than the rate for women without disabilities (32.8%) [7]. In addition, the rate of unemployment for men with disabilities (48.6%) is four times higher than the rate of men without disabilities (11.5%) [8]. Based on Figure 1, the outlier is Yemen, which has the lowest unemployment rate for women and men with disabilities (5.8 and 13.7%) [8].

Another difference is where they come from, whether from the urban or rural regions, because this has been shown to influence employability in the Kingdom and the Arab region in general. The unemployment rates for individuals with disabilities in rural areas of Saudi Arabia show that they are consistently the most disadvantaged cohort. In most countries in the Arab region, the unemployment rates and economic inactivity for individuals with disabilities are higher in urban locations compared to rural ones [9]. This is especially the case for females. This indicates that women with disabilities in urban locations, rather than in rural ones, experience marginalization [9]. Also, unemployment in the Arab region is mostly prevalent among the young urban middle class, while the poorest cannot afford to remain unemployed, opting to perform informal jobs [9]. However, there is a study that still insists that unemployment among people with disabilities still remains high in rural places [10]. While this is an issue of debate, different variables within urban and rural locations determine how people get employed, including those with disabilities, such as education, accessibility, level of discrimination, and immigration, among others [8].

### 1.2. Stakeholders/Actors and Their Roles in the Social Services Sector in the Arab Region

**Figure 2:**
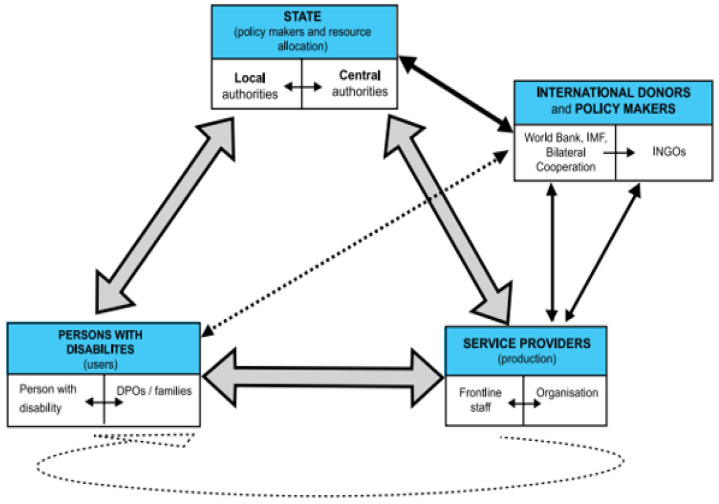
The actors in the social service system affecting people with disabilities in the Arab region [10]. The large grey arrows illustrate relationships among the three major internal stakeholders. The black arrows illustrate the relationships between the state and service providers (i.e., internal stakeholders) and the international donors and policy makers (i.e., external stakeholders). The dotted arrow between persons with disabilities (i.e., users) and international donors and policy makers illustrates a relationship in terms of monitoring, participation, and advocacy of their rights.

People with disabilities exist in families and societies that play a role in facilitating their well-being within the social services sectors, as illustrated in Figure 2. The main question is whether the different stakeholders are playing their roles effectively. If this is the case, then unemployment and discrimination would be minimal. For the wellbeing of people with disabilities to be met, the various actors must play their roles. These actors include the state (national and regional), the service providers (public, private, profit, non-profit, and religious), and the users (people with disabilities, their families and representatives) [10].

In the Arab region, culture, religion, history, and traditional gender roles are the predominant socio-economic situations influencing the stakeholders’ roles and responsibilities. Other actors include international developmental organizations such as the IMF and World Bank, and religious organizations that play a role in ensuring that the rights of people with disabilities are met. While there are several players, as illustrated in Figure 2, three stand out: the state, the service providers, and the users [10]. These groups define and execute the regulatory framework that the government needs to install for effective monitoring and control, and funding [10]. Table 2 provides the roles and responsibilities of different actors.

As reflected Table 2, each stakeholder has a role to play. As will be noted later in this study, one of the impediments hindering the success of people with disabilities is themselves, based on how they perceive themselves and partner with the non-disabled. This study assumes that, if all actors were to perform their roles, the well-being of people with disabilities, including their employability, would be safeguarded. 

## 2. Literature Review

The ability to work is an important component of successful living [11]. It is not only important as an economic activity but can also fulfil basic and social needs, contributing to and sustaining well-being and health [12]. For people with disabilities, work may be especially important because it can play a unique role in self-actualization insofar as it builds satisfaction and confidence that might not otherwise be available [12]. Consequently, employment for people with disabilities is an issue of global concern [12]. The fact that employment for people with disabilities is gaining the attention of practitioners and policy makers is important if one considers the proportion of the global population living with a disability [13]. Many countries are grappling with unemployment of people with disabilities [13]. For many people with disabilities, the challenge is not only access to employment, but also maintaining employment. This is a view supported by statistics from across the world [13]. For example, in the United States, 34% of the population lives with one or more disabilities and less than half are employed full-time [14,15,16]. In Canada, the employment rate for people with disabilities is 49% compared to 79% for those without disabilities [17]. In the European Union, the employment rate for people with disabilities is 47%, while the employment rate for people without disabilities is greater than 70% [18]. In Saudi Arabia, a survey of people with disabilities showed that, among those of working age, entailing individuals aged 15 years and older, more than 71% reported that they faced at least mild difficulties in securing employment [19]. The European Commission General Authority for Statistics reported that, although they have the right to work, the employment rate for people with disabilities is still not even one-half the rate of those who do not have disabilities [20]. Although the WHO reports that employment rates differ according to the country, worldwide, a person with disabilities has a significantly lower chance of securing employment than a person without disabilities [2].

A leading hypothesis for why employment rates are lower among people with disabilities is that employers may have negative views about the ability of such people to successfully perform the required tasks. This view is corroborated by a study [21] which concluded that employers had negative views in relation to people with disabilities. Several scholars report that these negative views are related to perceptions of skillset, intelligence, experience, productivity, and performance [22,23,24,25]. Studies conducted in Canada and the United States document how demographic factors such as education and experience may compound challenges for people with disabilities to secure employment (reviewed in [16]). Additional studies conducted in North America also identified effects of demographic variables on the rate of employment for people with disabilities [26,27].

People with disabilities have experienced discrimination throughout history, and the same applies currently; usually these individuals are viewed and treated differently and are often not accepted in the same way as their peers who do not live with disabilities [27]. To address discrimination, several countries have introduced legislation which aims to protect people with disabilities based on international agreements and regulations associated with preserving and protecting the rights of individuals with disabilities, guided by the principle of equality and with the goals of decreasing discrimination and promoting equal opportunities. Examples of these statutes include the Americans with Disabilities Act (ADA) of 1990 and the Official Development Assistance Act of 2005 in Canada. Notwithstanding these efforts, the ability of people with disabilities to secure and maintain employment is lower than that of their peers without disabilities [28,29]. In the US, the Covid19 pandemic helped tighten the labor markets and demand for remote working helped to increase the employment rate for those with disabilities [30]. The United Nations 2030 Agenda for Sustainable Development seeks to support and apply guiding principles for the achievement of progress that is sustainable, including promoting gainful employment for people with disabilities [31]. The agreement has been ratified by over 200 countries, including several in the Middle East [32]. In Saudi Arabia, endorsement and application of these principles have had desirable impacts on society, leading the Kingdom to oversee the employment of people with disabilities with greater success than many other countries, especially those in the Middle East [32].

Discrimination against people with disabilities in the Arab region is influenced by cultural, religious, historical, and socio-economic situations. One study on the employment plights of people with disabilities in Egypt found that people with a disability are often hidden from view, stigmatized, and denied work [33]. The same author investigates how disability affects women seeking employment, and he found that, if disability poses a challenge for women globally, then this doubled in the Arab world, since they have less access to employment opportunities than their non-disabled peers. Further investigation indicates that women experience less access to education, medical facilities, social life, and marriage in conservative societies where the status of women is relatively low, which creates challenges in daily life [33]. However, the situation has begun to change in recent years due to government initiatives and several NGOs, charities, and individuals raising awareness and integrating people with disabilities into daily life. In the Kingdom of Saudi Arabia, for example, the government is continuously endeavoring to provide a decent life for all citizens and residents, including those with disabilities, through legal frameworks [34]. The legal framework ensures that the rights of people with disabilities are met through the basic law of governance, protection from harm, social care, the provision of rehabilitation centers, health care, and equality in education [34]. Moreover, the legal frameworks being formulated also cater to issues like employment for those with disabilities, where employment initiatives are started that address mobility and transportation, facilities and parking, sign language support, and housing and mobile services, as well as opportunities to participate in decision-making concerning the King Salman Award for Disability Research [35].

Across the Arab region, two key dynamics typify disability rights movements: dynamism and fragility [34]. The United Nations Convention on the Rights of Persons with Disabilities (UNCRPD) hastened the regional recognition of disability as a focal point in human development and social justice initiatives [36]. Thus, new communication approaches, legislative mediation, and institutional capacity-building reflect present changes and innovation driven by grassroots initiatives led by or in partnership with disabled persons’ organizations [36]. The UNCRPD was applied in the United Arab Emirates (UAE) as a human rights model of disability to assess how the Emirates can meet its obligation towards people with disabilities in terms of higher education and employment [37]. Drawing from the experiences of people with disabilities in the Emirates and evidence from interviews done between 2016 and 2018, the findings show several main challenges to inclusion that stem from a weak enforcement and accountability framework that results in a lack of accessibility actions and support in universities and workplace environments [37]. The study recommended interventions that would create an institutional setting where people with disabilities are treated as rights-holders and given access to equal education and employment [37]. In many cases, people with disabilities are denied equal access to learning support and resources in universities and workplaces. One measure that was undertaken in 2010 by the UAE was to ratify the Convention on the Rights of People with Disabilities (CRPD), which gave people with disabilities rights to complain to the UN Committee regarding their rights and any violations of the Convention, laying a foundation for the inclusion of all learners in the UAE educational system [37].

### Summary

One of the limitations of the literature review is that it included much of the studies conducted in Western countries, with few studies done in the Arab region and, in particular, Saudi Arabia. The reason for this is that little literature exists about employment and disabilities in Saudi Arabia and the Arab region at large. While there are related studies on disabilities, they do not specifically address the issue of employment and the challenges that exist therein. Thus, this study was meant to fill this gap. Foreign organizations, including the United Nations, the World Health Organization, and the World Bank, have been cited. First, foreign countries were used to analyze what will be found in this study. This study assumes that, similar to Western and other foreign countries, people with disabilities are seldom employed compared with their non-disabled counterparts. This is a statistic that is shared around the world [13]. Studies from the US and Canada were included because most studies on disability and employability have been done there. Generally, the human rights recognition and acknowledgement in these two countries (the US and Canada), including for those with disabilities, is higher compared to the rest of the world. Therefore, these two countries make for better reference points in terms of providing recommendations for this study. Many of the mentions of these two countries involve their laws and efforts to address discrimination.

## 3. Methods

### 3.1. Design

The present study employs a qualitative, small-sample, case-report design. The data collection method is individual semi-structured interviews. This method made it possible for the participants to share personal perspectives in narrative detail. During the interviews, lasting about 35 min each, the questions asked were in relation to employment challenges and opportunities for people with disabilities. The interviews were recorded and transcribed verbatim. Participants were asked to clarify, correct, or add details during a review of the transcribed interview with the researcher. The qualitative methodology was selected because it affords detailed, rich, and personal responses. When designing the questions, the researchers’ aim was to determine how important employment is for people with disabilities, to bring to the fore the most important factors related to the employment of people with disabilities based on the perspectives of people with disabilities, and to identify potential obstacles preventing people with disabilities from securing or maintain employment in Saudi Arabia.

Second, qualitative research was suitable for the type of questions formulated for this study. An interview would be done as opposed to a survey, and this would allow for probing, which would allow us to get in-depth information about employment challenges faced by people with disabilities as well as what employers think about hiring people with disabilities. We had concluded that this was only possible through interviews and that questionnaires that would lead to statistical analysis, which would be limited in highlighting the problems faced by people with disabilities. Also, qualitative research generates rich, detailed, and valid data based on the respondents rather than our perspectives and interpretation of the issues investigated. It would also be possible to determine any variances amongst the respondents in their experiences and perceptions. These differences would be discussed clearly in an interview rather than a survey questionnaire. Lastly, qualitative research seemed practical for the number of participants we had in mind—10. We decided to target five employers and five employees who live with a physical disability. Quantitative research would have required a large sample size, which was not the case for this study.

### 3.2. Interview Questions

The specific questions presented to interviewees were as follows: Why is it important to hire people with disabilities, and what barriers and challenges are faced by these individuals? How is the employment of people with disabilities affected by demographic factors? How can people with disabilities overcome barriers, including discrimination, within employment settings?

The aim of this study was to identify the challenges linked to discrimination against people with disabilities in Saudi Arabia’s employment context. Another goal was to explore possible strategies that may be executed to promote equal opportunities and create an inclusive work setting. The questions formulated (as shown above) touch on these matters specifically. The questions have also been formulated to allow for probing by asking multiple questions concerning the same thing, be it challenges or overcoming barriers. The questions were kept to a minimum to focus on specific questions relevant to this study. The questions also address other goals, such as providing recommendations based on the experiences of people with disabilities by asking how they can overcome barriers.

### 3.3. Participants

Ten men participated in the current research. These included five men with disabilities and five employers, all residing in Saudi Arabia. The sample was selected using convenience sampling and word-of-mouth. The demographic details of the participants are presented in Table 3 The ages of the participants ranged between 25 and 45 years. They had varied levels of employment experience, ranging from 4 to 20 years. Participation was voluntary and not rewarded. Although the respondents were informed that they could withdraw from the study at any point without reason or penalty, no participant withdrew from the study.

#### 3.3.1. How Disability Was Operationalized

This study targeted respondents with a physical disability even though disabilities exist in more than one form, such as cognitive disability. Generally, disabilities can be behavioral/emotional, a sensory impaired disorder, physical, or developmental [38]. The reason behind this choice of physical disability is that it is more apparent than all other forms of disability; hence, these individuals are likely to have experienced obvious bias and discrimination. We argued that other types of disabilities, such as behavioral and sensory disabilities, can be assumed or ignored, but not physical disabilities. We particularly focused on people who could not walk properly (had to use a wheelchair or walking crutches) and were blind and deaf. These three forms of physical disabilities tend to be considered burdensome for others—and this would be the case in a work setup as well. Moreover, employers would require additional resources to assist people with physical disabilities such as an inability to walk, blindness, or deafness. For instance, an organization would need pathways, parking, and restrooms for people who cannot walk normally. Furthermore, visually impaired individuals need braille scripts and other assistive technologies, such as voice recognition software and augmentative communication devices, to communicate well in the workplace [39]. This criterion would determine the finding that employing people with disabilities can be costly, as supported by another study [40]. For this study, three participants had a walking disability; one was blind; and the other was deaf.

#### 3.3.2. Use of Male Participants Only

The study consisted of male participants only—both the employers and people with disabilities. This is because they were the only ones available at the time of the research—convenience sampling was used. Moreover, we had assumed that females with disabilities seldom get employed, and few employers are females compared to males. As seen in Table 3, the participants of this study—whether employer or employed—work in private organizations with a high male population compared to females. In other words, this study has divulged that there are few female employers in the private sector and few females living with disabilities who are employed. This is consistent with one of the findings discussed later that indicated females with disabilities experience more discrimination than their male counterparts both at work and in general [41]. Moreover, one of the limitations that will be highlighted later is that the participants with disabilities did not divulge as much information as would have been expected, either due to embarrassment or shame. We assumed that this would have been worse had women participated and decided to do the study with male participants without needing women. This is a limitation for the entire study since we could not determine the view of females and, thus, provide balanced information about employability between both genders.

### 3.4. Procedure and Materials

Ethical approval was obtained from King Abdulaziz University by the Scientific Research Ethics Committee of King Abdulaziz University (protocol 4527929, date of approval: 3 October 2023). Prior to the interview, participants reviewed and signed a statement of informed consent. Privacy and confidentiality of participants was guaranteed by the interviewer. A digital device was used to audio record each interview. Once the interview (conducted in Arabic) was completed, the interviews were transcribed verbatim and then translated into English by a local translator. Themes emanating from participants’ responses were identified based on mixed descriptive and inductive methods designed for this purpose and successfully used by the current research team in a previous study [41].

### 3.5. Data Analysis

As mentioned in the previous section, the interviews were transcribed verbatim and translated into English. The sources were marked, and their demographic attributes were noted (codes were provided, as shown in Table 3). The next step was to read the information which was provided—this was done several times as a research group. We also kept notes about our thoughts and any questions regarding the data to ensure that the data were appropriate. We later used highlighters and concept maps to connect the data. We noted the keywords and phrases and made notes in the margin to group the data. This was followed by analyzing the data to identify recurring themes, opinions, and beliefs amongst the employers and employees with disabilities. Themes were identified by checking similarities between key words and phrases among different participants’ information. These themes were later used in the Results and Discussion sections. Lastly, we considered the study’s purpose, which influenced the content/wordings that were included in order to tell the narrative of the data best. 

## 4. Results

### 4.1. Why It Is Important to Employ People with Disabilities

During the interviews, all participants—both people with disabilities and prospective employers—voluntarily commented that employment for people with disabilities is important because it provides an opportunity for all parties to contribute to building a more inclusive society. All participants from both groups also voluntarily endorsed views consistent with a social justice perspective, noting that, when people with disabilities are gainfully employed, they can make an effective contribution to the labor market. In highlighting this point, employer E1 explained:

The idea of disability should not be perceived to mean that we should pay more attention to the disability. Rather, it should mean that we pay more attention to the abilities of the individual living with a disability, together with the strengths such an individual possesses. Work is especially vital for people living with disabilities because it increases their satisfaction and confidence and independence and self-sufficiency.

This view was shared by employer E2, who said: “Nothing is more important than work for those living with disabilities specifically. This is because work provides them with freedom, independence, and social participation, and a decent life that most dream of”. The importance of work for people with disabilities was also noted by employer E4, who stated that employment constitutes a necessary factor in the lives of people with disabilities because it “helps them to be independent, provided that they are able to overcome the challenges they face when attempting to assume responsibility and live normal lives”. In agreement with this view, employer E5 said, “When people with disabilities are employed, their sustainability is enhanced as doors of opportunities also open”.

### 4.2. Employment Challenges for People with Disabilities

All five participants with disabilities reported that few employers view them in a positive way. They all commented that, when prospective employers look at them, they focus on what they cannot do instead of what they can do. This would make the participants with disabilities feel unwelcome in the workplace, in the event that they were hired. Participants with disabilities reported that they must deal with obstacles and challenges, both before they are employed as well as once they are employed. For example, all participants with disabilities commented that, once they are employed, they must contend with discrimination and bias, which can have a negative effect on their ability to work, as well as on their commitment, productivity, performance, health, and psychological well-being. The following sections provide additional detail about these employment challenges.

False perceptions. The perceptions of many employers regarding people with disabilities are inaccurate. According to the participants with disabilities, this can lead to discrimination and bias in hiring and, if they are hired, to discrimination and bias in the workplace. Generally, according to the participants with disabilities, the idea of employing people with disabilities is still not well accepted. This is made worse by the fact that there is limited awareness in society regarding issues relating to people with disabilities. According to the participants with disabilities, negative views among employers in relation to people with disabilities are based, in part, on the employers’ view that they are too expensive to hire and require constant monitoring. This means that employers often do not trust the skills and abilities of people with disabilities. According to the participants with disabilities, when employers hire people with disabilities, they are concerned that these employers do so out of mercy and kindness, rather than because they have the appropriate skillset, education, and experience.

Ineffective laws. Although laws have been enacted in Saudi Arabia to facilitate the employment of people with disabilities, these laws have tended to be relatively ineffective, according to the participants with disabilities. Within this context, many employers view people with disabilities as unreliable, unable to work, non-compliant, prone to be late, and often absent from work. Employers may also believe that people with disabilities will have an adverse effect on their colleagues. This can lead to these individuals being marginalized in the workspace, with many colleagues preferring that their workplace did not have people with disabilities. Employees without disabilities view their counterparts with disabilities as a burden, according to the participants with disabilities.

Lack of training opportunities. According to the participants with disabilities, opportunities for education and training are often not available for persons with disabilities. This makes it a challenge for them to enhance their skills and abilities so that they can be more attractive employees. This results from a lack of awareness and knowledge among employers regarding the capabilities of people with disabilities and how they can contribute to the labor market. The result of this lack of awareness and knowledge about people with disabilities is a decrease in their opportunities, which implies that some employers do not have adequate experience and need to be trained in suitable methods for dealing with people with disabilities. In Canada for instance, Canada’s Disability Inclusion Action Plan 2022 is not only creating employment strategies for people with disabilities but also providing training to help them advance in their careers as well as causing some of them to become entrepreneurs [42]. Thus, if people with disabilities cannot become employed, their skills can be utilized to create employment.

Participants with disabilities commented that many workplaces and their facilities cannot easily be accessed by people with particular physical disabilities (e.g., confined to a wheelchair, not able to navigate stairs into a workplace). Another impediment is the failure to provide access to software and assistive technologies to make it easier for people with disabilities to participate fully in the work environment. In relation to the points raised above, employer E1 stated.

Many people living with disabilities have good experience and skills and can perform well if they are provided with the opportunity and job suitable for their kind of disability. In most cases, when people with disabilities fail to live up to certain standards, the explanation could be a number of elements and challenges linked to how compatible the job is with the disability and the accessibility of facilities in the workplace. For instance, it may take more time to do tasks involving typing for a data clerk who has arthritis but does not have an ergonomic keyboard or mouse. In such a case, the performance of such a clerk may be deemed as lower when compared to colleagues without disability who can use a standard keyboard and mouse.

In the same vein, employer E3 commented: “In most cases, it is challenging for us as employers to understand the feelings and needs of people with disabilities. I could say that this can be attributed to the fact that these individuals do not communicate clearly. This can present challenges in communicating and exchanging information, and at the same time there are many people with disabilities.” Employer E4 agreed with this sentiment, noting: “Because people with disabilities are often unable to fairly compete in the labor market, these individuals are often uneducated and unqualified. When they get jobs, they may find it challenging to understand the tasks they are assigned and also in dealing with their colleagues.” 

### 4.3. Impact of Demographic Factors on Employment Challenges for People with Disabilities

There was a general agreement among all participants—both those with disabilities as well as prospective employers—that demographic factors can have an important impact on the employment landscape for people with disabilities. Gender, age, disability type, and education can limit or shape the opportunities available to people with disabilities. A more detailed discussion of these factors as reported by the participants is presented below. 

Education. Employer E5 commented that, “For people with disabilities, education has an important role to play. With an education, it is easier to enhance skills and abilities, which increases the chances of finding an appropriate job.” Employer E4 noted that, “Employment outcomes can be affected by access to quality education. When education opportunities are limited, the result is a lack of skills needed for employment.” Participant with disability E1 offered that, “In today’s skills-based occupations, work is becoming more specialized. Therefore, levels of education among people with disabilities may impact their ability to work and assimilate into the environment.” Echoing this sentiment, participant with disability E10 stated that, “For people living with disabilities, one of the biggest challenges we face is that we are not appropriately educated. Even where we are granted educational certificates, some official authorities refuse to recognize them”.

Disability type and age. Participants with disabilities as well as employer participants commented that the disability type and age can have important effects on the employment landscape and career growth opportunities for people with disabilities. Participant with disability E7 noted that, “The abilities and needs of people with disabilities differ, depending on the kind of disability, which impacts the actions and tasks that can be done in the workplace.” Employer E3 noted that, “The process of recruitment can be impacted by the type of disability. Certain jobs may call for specific techniques and skills depending on the kind of disability, and the challenge can be mitigated by paying attention to evaluating skills and abilities, notwithstanding the type of disability in question.” Employer E8 stated that, “Employment rates decrease with age, and the employment gap tends to be higher as the ages get higher, and this phenomenon is clearer among people with disabilities. I see this as showing the interface between living with disability and age.” Employer E1 noted that, “Individuals with disabilities experience greater barriers, and age can have an impact on their employment. In many instances, they may have special skills and experiences. This could make them stronger in the work environment. However, they may face difficulties in other areas. I believe that it all depends on the type of disability and age. I think that as part of creating awareness around this matter, employers need to act in a different way when dealing with people with disabilities, considering that the appearance of many people with disabilities will change throughout the year and that individual’s life and career may also change as they do. For example, the severity of the disability may increase if the individual develops new disabilities.”

Finally, employer E4 offered that, “When it comes to employing people with disabilities, age may play a role because it can impact their disability and experience. The fact that younger people may face educational barriers could emphasize the significant role played by assessing the skills of individuals and their competencies to make sure that there are equal opportunities for employment”.

Gender. Regarding the impact of gender on employment prospects, especially for people with disabilities, the participants offered thoughtful comments expressing their views. For example, employer E3 noted that, “The challenges and opportunities faced by women and men with disabilities in the development of their career paths and recruitment process differ.” According to employer E2, “Gender may have an impact on employment chances for individuals with disabilities. These people may face added problems as a result of gender discrimination. It is for this reason that nondiscrimination and fair employment policies should be encouraged. In this regard, it is vital to promote awareness and understanding of equality and diversity within the workplace.”

Employer E1 stated that, “The burden for women is double, but to employ women with disabilities, just like men, calls for paying attention to their skills and abilities as opposed to their disability”.

Overcoming workplace barriers. In terms of overcoming workplace barriers, employers E1 and E2 shared the view that, earlier in their careers, they assumed that individuals with disabilities would perform poorly in their duties. They noted that they expected greater delays and absences from work. However, they also noted that, with time, they realized that their assumptions were incorrect and that people with disabilities had capabilities that matched those of their colleagues without disabilities. E6, a participant with a disability, noted that, “It is vital for employers to be clear about all the abilities needed to perform a specific task right from the beginning of the application process. A job analysis should be employed when determining the need for these abilities, and the main focus should be on abilities as opposed to disabilities”.

All five participants with disabilities noted that workplace discrimination can be averted through the promotion of awareness and adoption of inclusive policies, ensuring that there are reasonable accommodations for people with disabilities in the workplace, and ensuring suitable technology for facilitating the performance of tasks. The participants shared the view that, when employers are educated, it becomes easier for people with disabilities to work on an equal footing with their peers without disabilities. According to these participants with disabilities, educating the employer makes it easier to protect the rights of people with disabilities so that they receive equal pay for equal work. This is an important point, considering that it is still common for people with disabilities to receive lower pay for the same work as their colleagues without disabilities. Added to this, the participants referred to the reality that there are inadequate incentives and promotions which establish an accessible, healthy, and safe environment. The participants with disabilities added that people with disabilities need to accept that they may play a role in the discrimination they face, and that they can play a part in breaking down the barriers. These participants commented that securing and maintaining employment is more likely with training, education, and the acquisition of applicable skills and qualifications for enhancing competitiveness in the labor market. E7 and E8, participants with disabilities, noted that the challenges linked to discrimination and bias in the workplace call for inclusive strategies and the establishment of fair and transparent employment policies for people with disabilities. To achieve this, it is important to educate both employers and employees, because awareness is crucial for improving workplaces and changing stereotypes.

E9, a participant with a disability noted that, “Programs geared at training employees should be developed with the aim of creating a work environment that nurtures diversity and respect for individuals without regard to their physical ability”. Consistent with this view, E10, another participant with a disability, noted that, “It is important to train employees with regard to how they should approach people with disabilities, regardless of their health condition, so that the basis of work is mutual appreciation and respect”. Employers E3 and E4 agreed that, to dismantle the impediments leading to discrimination in the workplace, it is vital that people with disabilities develop their abilities and skills with regard to the use of assistive technology to change negative stereotypes and show that, behind the work they do, there is willpower and the ability to succeed, even though they face challenges.

## 5. Discussion

Gainful employment nurtures individual well-being and social sustainability [43,44]. For people with disabilities, gainful employment provides opportunities that boost not just their economic sustainability but also their social participation [44]. This is a view also expressed by [45], which posits that, when organizations employ people with disabilities, they contribute to those individuals’ independence, providing them with opportunities for interactions with co-workers, feelings of social support, personal acceptance, and social participation. The same scholar notes that gainful employment of people with disabilities helps to build a society that respects and values a diversity of abilities and makes it possible for people with disabilities to be self-reliant, which can produce positive changes in their lives. Relatedly, work is an important element in the lives of people with disabilities because it assists them in building their personal identity and finding meaning in their lives [46]. The same authors conclude that work is a pivotal element in determining if people with disabilities live satisfactory or frustrating lives. Consequently, the effect of employment on the quality of life for people with disabilities corroborates the argument that people with disabilities should be given access to employment [47]. This view is in agreement with the views expressed by all the participants in the study, who noted in various ways that the spirit of providing employment to people with disabilities lies in establishing a society that values diversity and is willing to invest in it, which in turn contributes to social solidarity and leads to progress that includes everyone. More generally, participants with disabilities and employers interviewed for the current study agreed that employment for people with disabilities is important because it increases their independence and self-sufficiency and, in turn, their satisfaction and confidence.

Saudi Arabia is currently experiencing social and economic transformation, and ensuring that people with disabilities have access to gainful employment is important in achieving the goals identified by the Kingdom’s Vision 2030. The Disability Welfare Act sets out the legal framework for the promotion and protection of the rights of persons with disabilities in Saudi Arabia [7]. Vision 2030 incorporates plans and programs whose aim is to make Saudi Arabia a model that other countries in the Middle East can emulate, including in the area of ensuring that people with disabilities have suitable job opportunities and educational opportunities that facilitate their independence and integration into society so that they can function as contributing members. Vision 2030 seeks to ensure that all Saudi Arabian citizens have access to the tools and facilities that will assist them in achieving success. Making employment available to people with disabilities in Saudi Arabia is, therefore, important if the Kingdom hopes to create an inclusive and just society, as outlined in Vision 2030 [7].

### 5.1. Challenges for People with Disabilities

It has been noted by the WHO that 15% of the people in the world live with a disability, representing about one billion people [2]. With rising chronic diseases and life expectancy, natural disasters, violent conflict, forced migration, and birth defects are expected by the United Nations to increase the numbers of people with disabilities [48]. What makes disability a particularly complex challenge is that it has the effect of limiting the performance of daily activities and the ability to integrate successfully into society. If one considers that a lack of employment has negative effects on a person’s psychological and physical well-being, it is clear that unemployment is detrimental to people with disabilities and the communities in which they live [49]. Added to this, people with disabilities face challenges relating to working in a manner that allows them to live a dignified life. Apart from facilitating their gainful employment, organizations need to ensure that people with disabilities feel accepted and welcomed [50]. Where an organization fails to facilitate and maintain a welcoming atmosphere, an individual with a disability may feel useless or burdensome, which leads to a loss of confidence. This view is corroborated by the reports of participants with disabilities interviewed for the current research, who noted that they often feel unwelcome and that they are viewed negatively by prospective employers, suggesting that employing people with disabilities is still unacceptable in many workplaces.

Some employers assume that employing people with disabilities is too costly. Other employers appreciate the value of people with disabilities, seeing them as productive and valuable team players [50,51]. Numerous factors affect whether employers view people with disabilities negatively or positively, including likely acceptance by co-workers, necessary skills, experience, commitment, productivity, performance, and the anticipated costs of employing them [48,49]. Among the primary reported concerns of prospective employers is that people with disabilities will not be as productive as their counterparts who do not have disabilities [52,53]. Related research reports that people with disabilities are perceived to be slower in performing their work and more likely to take leave days, leading to tardiness and a higher rate of absenteeism [50]. In some cases, employees with disabilities are viewed as lacking commitment to their job. A study about the satisfaction of employers with employees who have a disability concluded that there are differences in employer assessments of employees with disabilities and employees without disabilities [4]. Specifically, employers report that they are less satisfied with the performance of individuals with disabilities in comparison to their peers without disabilities. Employers also noted greater concerns related to the speed, accuracy, performance, and cost of employees with disabilities relative to employees without disabilities [21]. Corroborating this research, a study concluded that employers are reluctant to hire people with disabilities even when they have the relevant experience and qualifications [54]. The results of the current study corroborate these findings. In the current study, however, several employer participants reported a positive valuation and perception of people with disabilities, indicating that they treat them according to their abilities to perform the work. Indeed, several employer participants in the current study explicitly commented that attention and focus must be paid to the individual’s abilities and strengths rather than their disabilities.

### 5.2. Demographic Factors

The World Bank notes that the employment opportunities and social status of people with disabilities vary between demographic groups defined by education and age [55]. A wide-ranging study focusing on the impact of demographic factors, work experience, and use of disability employment services on people with disabilities was conducted by [56,57,58]. The results indicated that age, gender, disability type, and education each affect a person’s functional status. This is consistent with the results of other studies [59,60]. This body of work suggests, for example, that employment outcomes improve with additional education, which leads to a narrowing of the employment gap for individuals with disabilities [59]. Theories based on the assumption that demographic and disability variables affect employment during developmental maturation have been presented previously [58,59]. The reports of participants in the current study—including employers and people with disabilities—corroborate the results of previous research insofar as demographic factors are identified as having important effects on the employment landscape for people with disabilities, limiting or otherwise impacting the opportunities available to them. We discuss these variables in the following sections.

Education. Previous research indicates that education is among the most important determinants of securing and maintaining gainful employment for people with disabilities. Studies in Canada and the United States document how education affects employment among individuals with disabilities and that demographic factors are often associated with employment outcomes [16,17], respectively. In the United States, the employment rate for people without a disability who are 25 years or older and who do not have a high school diploma is 54%, whereas for those with a bachelor’s degree it is 76% [61]. The same applies to people with disabilities, although the employment rates are significantly lower. The employment rate for people with a disability who are 25 years or older and who do not have a high school diploma is 33%, whereas for those with a bachelor’s degree it is 69% [61]. A study conducted in Canada found that the percentage of employed high school graduates with disabilities was lower than that for people without disabilities [62]. The participants in the current study—both people with disabilities and employers—offered comments that confirm the results of this previous research, highlighting the importance of education and its impact on the employment of people with disabilities.

Disability and age. The impact of environmental, social, and physical factors on the opportunities for employment for people with disabilities has been assessed [63]. These factors include the nature and severity of the disability and the general health of the individual. It has been concluded that, generally, people with a more severe disability face more obstacles in securing and maintaining employment [63]. People with physical disability often explicitly report concern that their disability will make it challenging for them to perform tasks that are physically demanding [64].

Participants in the current study—both people with disabilities and prospective employers—stated that the recruitment process can be influenced by age and disability. As was stated by one employer participant in the current study, employer awareness of the challenges faced by people with disabilities and that these challenges may vary with age will need to be increased to ensure equal employment opportunities for people with disabilities, especially with regard to changes over a lifetime. This view is supported by a study that suggests that the skills and limitations of people with disabilities may change over time, affecting their ability to perform work-related tasks [65]. In addition, depending on the severity of the disability, the possibility of hospitalization escalates with age [65]. This means that an employee who begins a job without a functional disability could develop a disability, and this is also true for people with and without various disabilities [65].

Gender. Although the situation is improving, there is evidence of discrimination in the workplace against women in Saudi Arabia and, indeed, worldwide, we find that women with disabilities who work in the workplace suffer from discrimination [47]. When women have a disability, this discrimination can be greater than it is for men with a disability. Globally, there is evidence of employment inequities which persist between men and women with disabilities [66].

What the participants report in the current study confirms the results of previous studies that education plays an important role in job opportunities, and participants noted that this is especially so for women with disabilities. They also noted that the kind of disability an individual lives with, together with suitable vocational training, may affect job opportunities. This suggests that it is important to support young people with disabilities—perhaps especially women—at the beginning of their careers. In recent years, Saudi Arabia has acted to reduce employment obstacles by focusing on these demographic factors (especially education, but also age and gender) to ensure that people with disabilities are fully integrated into a society that values diversity. From a practical perspective, this is achieved by broadening educational opportunities for people with disabilities while also providing professional educational initiatives to develop their skills. Saudi Arabia has also focused on legislation to advance and support the rights of people with disabilities, especially women, and to reduce discrimination against them. Sustainable employment for people with disabilities requires a focus on challenges and opportunities for men and, especially, women with disabilities.

### 5.3. Addressing Employment Discrimination

Although there have been many efforts and strategies which aim to promote equal employment opportunities and reduce discrimination against people with disabilities, there remain significant gaps in employment and participation of these individuals in the workplace [67,68]. And the employment rates of people with disabilities are lower when compared to those of their counterparts without disabilities. Corroborating this literature, participants with disabilities in the current study commented that, when they are able to secure employment, they typically discover that their pay is lower than the pay of people without disabilities.

The global community is steadily becoming aware of unequal opportunities for people with disabilities. This has led to global efforts such as Article 27 of the Convention on the Rights of Persons with Disabilities, Committee on the Rights of Persons with Disabilities (2016), and the 2030 Agenda, which states that people with disabilities should have the right to the opportunity to earn a living by work freely chosen or accepted in a labor market and a work environment that is open, inclusive, and accessible to all [69]. This statement shows that the right to equal pay for equal work remains an objective to be met for people with disabilities. Participants in the current study—both participants with disabilities and prospective employers—commented that, if barriers linked to discrimination are to be removed, one area that must be addressed is providing sufficient opportunities for people with disabilities to secure skills and experience through education and job training. In addition, most participants noted that employers must consider the needs of individuals with disabilities within the work setting in relation to designing facilities and buildings to facilitate access, and providing appropriate assistive technologies for people with disabilities so that they can successfully perform. Participants further noted that organizations representing people with disabilities and those representing employers should advocate for the enactment of relevant legal statutes that will ensure a safe and healthy work environment for people with disabilities, from recruitment to promotion, such that each individual is protected from discrimination.

An example of such statutes is the 1990 Americans with Disabilities Act (ADA), which prohibits discrimination against people with disabilities in several domains, including employment [70]. Businesses are required by this law to provide accommodations that facilitate access for people with disabilities and ensure that the rights of people with disabilities are protected at work. Canada unanimously passed the Accessible Canada Act (ACA) in 2019 and, in addition, the province of Ontario passed the first law of its kind, called the Accessibility for Ontarians with Disabilities Act (AODA), in 2005 [71]. The AODA outlines a plan for Ontario to become fully accessible for all people by the year 2025. Canadian law makes it an offence to discriminate against individuals with disabilities in areas of employment and stipulates that companies must ensure that people with disabilities can access the work environment [71]. Through its 2030 Agenda for Sustainable Development, the United Nations (2021) aspires to alter the expectations of and attitudes toward individuals with disabilities. The 2030 Agenda encourages nations to take practical measures to bring to the fore the challenges faced by people with disabilities and ensure that they are included in policymaking [65]. In the United Kingdom, wide-ranging initiatives have been enacted to reduce the discrimination experienced by people with disabilities. The relevant law is known as the Equality Act and promotes equality regardless of age, disability, or gender [72]. In Saudi Arabia, the law requires that people with disabilities have the right to enjoy the highest degree of social care, while the state accounts for the moral and material needs of people with disabilities while also seeking to ensure such individuals exist in an environment that is comfortable. This is facilitated by establishing training programs to develop the skills of people with disabilities to thereby increase their employment opportunities.

Corroborating the sentiment of these statutes, participants in the current study suggested that the government should act to increase awareness of the challenges faced by people with disabilities in Saudi Arabia, perhaps through media campaigns, to alter the prevailing perceptions of individuals with disabilities, and enact policies and laws to ensure that people with disabilities and their rights are protected. In addition, participants in the current study noted that employment discrimination could be addressed by providing incentives and benefits to organizations to employ appropriately qualified people with disabilities. An important practical step in this direction was the signing in 2020 of the Saudi Arabian Welfare of Persons with Disabilities and the Human Resources Development Fund (2019) [73]. The aim of this signing was to facilitate training and employment initiatives that could positively enhance the empowerment and employment of people with disabilities [73]. Based on the comments offered by participants in the current study, we suggest that employment efforts should be broadened with proactive partnerships between organizations, professional agencies, and community-based entities specializing in supporting the employment requirements of people with disabilities.

Aligned with this framework, higher education institutions play a pivotal role in advancing the employability and personal development of those with disabilities, who might otherwise find such educational opportunities out of reach. There is an increasing number of specialized programs designed to bring individuals with disabilities into the fold of higher education around the world. These programs are structured to provide a broad range of skills that are essential for academic success, engagement in social services, workforce participation, and fostering independence and self-determination [74]. These educational initiatives support and extend the goals outlined by Saudi Arabian efforts, together forming a comprehensive approach to improve educational and employment outcomes for individuals with disabilities.

## 6. Conclusions

Participants in the current research—including prospective employers and people with disabilities—agree that employment is not only a means of securing income to live independently, but also a mechanism for increasing and maintaining a sense of confidence and self-esteem, which is important for the success of individuals, regardless of their disability status, and the successful functioning of society [75]. It is also clear that most people with disabilities can be successfully trained and employed. When they are afforded employment opportunities, people with disabilities can contribute to society just as surely as people without disabilities. Thus, people with disabilities should not be perceived as a burden on society.

The goal of the present study was to explore the role played by employment in the lives of people with disabilities in Saudi Arabia, where the challenges they experience have been documented in previous work. The current study considered how demographic factors can impact employment opportunities for people with disabilities in Saudi Arabia and how these opportunities can be increased. The current study also considered the impact of discrimination in the workplace and addressed the means by which discrimination can be reduced. The aim of this analysis was to provide initial guidance on improving the employment opportunities and environments for people with disabilities.

Providing employment to people with disabilities in Saudi Arabia contributes to the attainment of diversity and social inclusion. The present study’s results also illustrate the impact of demographic factors including previous experience, age, gender, and education on the likelihood of employment. The insights suggested by the reports of participants in the current study indicate that workplace discrimination can be reduced by the promotion of awareness and by implementing initiatives and policies which encourage equality and equal opportunities for people with disabilities.

### Limitations and Recommendations

One of the limitations of this study is that the perceptions of participants in the present study may be limited because they depend on the specific lived experiences of this small sample of individuals, all of whom were adult men. Also, participants may, for various reasons, have been reticent to divulge personal information and only shared some of their experiences while deciding not to talk about others.

Based on the insights obtained from this study, it is recommended that there should be efforts to secure a better understanding of the importance of employing people with disabilities. It is important to bring to the fore the obstacles that hinder employment opportunities for these individuals so that recommendations can be made regarding what needs to change to accommodate such individuals. Insights relating to how to enhance employment opportunities can facilitate and support suitable vocational training and education. Moreover, greater awareness of the employment challenges faced by people with disabilities in Saudi Arabia could facilitate the adoption of institutional policies which promote equality and decrease discrimination in the workplace. Also, as noted in Table 2, certain stakeholder and actor groups exist to cater to the needs and well-being of people with disabilities. If these groups performed their roles and responsibilities effectively, then the rights of people with disabilities would be upheld, and discrimination would decrease. The recommendations herein will provide more information on how these actors can improve to ensure that the rights of people with disabilities are met. Therefore, based on the results obtained from the findings and other information noted earlier in this study, we offer the following recommendations:

In general:

- Service users and disabled people’s organizations (DPOs) continue to claim their rights to the authorities and influence policymakers. Additionally, users should be enabled to participate in service provision, receive sufficient information, and raise complaints concerning the quality of the services provided; as well, they should be able to choose the types of services provided. The participation of DPOs as users should enable them to monitor and assess the processes of state authorities, which do not always generate satisfactory outcomes.
- The state, through the central and local authorities, has a role in evaluating the needs and demands of individuals with disabilities for social services and ensuring their accessibility and availability—this can be done through the collaboration of all stakeholders. However, the DPOs, as mentioned earlier, should claim their rights to the authorities and ensure that they are supported. The state can also ensure that this happens through sufficient financial programs. Social services can be availed through public service providers, such as in the education sector or hospitals, or by delegating the responsibilities to private and non-profit organizations. This happens when hospitals operate, and civil society groups can provide social services. The state, however, should maintain the role of regulation and be able to define adequate policies for executing laws and comply with international laws it has signed.
- Service providers should be responsible for providing quality services based on a person-centered and inclusive approach that complies with defined quality standards. All stakeholders should agree on what meets quality principles and standards. Also, service providers can engage in the tendering processes and, at times, be eligible to receive public funds to help those with disabilities. Nonetheless, service providers should also recognize that users have the right to express their demands, participate in or initiate the definition of their needs and individual plans, and be involved in phases of strategic planning to monitor and assess effective service delivery.

At the work place:

- Provide appropriate training and education for people with disabilities, in keeping with their abilities, so that they are adequately prepared for the labor market. This can be achieved through the development of academic programs and study plans, developing initiatives, and the consideration of modern trends to better meet the needs of the labor market.
- Provide training and education to employers and colleagues of people with disabilities in relation to how to treat people with disabilities. This could also involve increasing awareness of the lived experiences of people with disabilities in relation to discrimination and stereotyping.
- Support the activities of various agencies to ensure adherence to existing statutes intended to establish job opportunities for people with disabilities. Also, it should be noted that, for laws to be used effectively to deal with discrimination, the laws should be supported by the employers and not forced on them.
- Ensure the availability of modern devices and technologies to support the success of people with disabilities in their place of work.
- Utilize media as an important resource for increasing community awareness of the employment challenges of people with disabilities in Saudi Arabia.
- To manage disability in the workplace, employees with disabilities should be reviewed and appraised. The performance appraisal of workers with disabilities should take place according to the same criteria applied to holders of the same or similar jobs.
- Employees with disabilities who show superior skills and are interested in engaging in entrepreneurship should be encouraged to do so and mentorship programs should be accorded to them just like the non-disabled. Linking employees with disabilities with successful entrepreneurs who have disabilities can show them that they too can be successful in entrepreneurship.
- Employees with disabilities should be encouraged to seek promotion, especially if it appears that they are hesitant to do so due to a disability-related impairment or other difficulty, or perceived hurdles in their working environment.
- Opportunities for workers with disabilities to participate in in-service training programs should be created and promoted. Where necessary, competent authorities or organizations should enable the use of readers, interpreters, and adapted materials to support people with disabilities.

## Figures and Tables

**Table 1:** Type of disability and the number of individuals with the disability in the Kingdom [7].

Disability Type	Number
Hearing Impairment	289,355
Attention-Deficit/Hyperactivity (ADHD)	30,155
Mobility Impairment	833,136
Autism Spectrum Disorder (ASD)	53,282
Down Syndrome	19,428
Visual Impairment	811,610

**Table 2:** Roles and responsibilities of different actors [10].

Stakeholder	Roles and Responsibilities
State Central Authorities	Ensuring fundamental rights are met. Regulating social services. Distributing resources and funding social services. Assessing demand at the macro-level and planning of services. Monitoring quality. Ensuring availability and quality of professionals.
Local Authorities	Providing social services/delegating and contracting out. Assessing demands at the local and regional levels. Coordinating local planning and ensuring adherence with the national laws, policies and strategies. Ensuring participation of people with disabilities. Evaluating quality. Financing projects.
Social Service Providers Public and private providers Profit and non-profit organizations Religious organizations Informal providers (families and volunteers)	Ensuring quality services are provided. Evaluating individual demands. Providing services and adequate information. Promoting positive images to the public to reduce discrimination. Adapting to users’ needs. Ensuring users’ rights are met and their active participation.
Users of Social Services Disabled People Organizations (DPOs) Individuals with disabilities Families/and legal representatives of individuals with disabilities	Right-holders with responsibilities. Participate in service planning and provision, monitoring and assessment. Represent users and advocate for their rights, including the creation of adequate services for individuals with disabilities.

**Table 3:** Participants demographics (*n* = 12).

No	Code	Age (Years)	Years of Work Experience	Sample Classification	Organisation
1	E1	45	20	Employer	Private
2	E2	44	18	Employer	Private
3	E3	41	15	Employer	Private
4	E4	39	13	Employer	Private
5	E5	37	12	Employer	Private
6	E6	34	9	Person with disability	Private
7	E7	33	7	Person with disability	Private
8	E8	31	5	Person with disability	Private
9	E9	27	4	Person with disability	Private
10	E10	25	2	Person with disability	Private

## Data Availability

Due to the nature of this research, participants did not agree to having their identified data be publicly shared.
